# Amplitude-Phase Variation in a Graphene-Based Microstrip Line

**DOI:** 10.3390/mi13071039

**Published:** 2022-06-30

**Authors:** Muhammad Yasir, Sergej Fatikow, Olaf C. Haenssler

**Affiliations:** 1 OFFIS Institute for Information Technology, 26121 Oldenburg, Germany; muhammad.yasir@offis.de; 2 Division of Microrobotics and Control Engineering, Department of Computing Science, University of Oldenburg, 26129 Oldenburg, Germany; sergej.fatikow@uni-oldenburg.de

**Keywords:** graphene, attenuators, phase shifters, modulators, passive circuits

## Abstract

A graphene-based transmission line with independent amplitude and phase variation capability is proposed. Variation of graphene’s tunable conductivity by an applied DC bias is exploited in designing an attenuator and a phase shifter. The attenuator and phase shifter are separated from each other by an interdigitated capacitor to ensure independent control of each section through an applied DC bias. The phase shifter is designed by optimizing lengths of a tapered line and an open stub for a maximum variation of input reactance with a change in graphene resistance. The attenuator is designed by two pairs of grounded vias connected to the transmission line through graphene. Variation of graphene resistance controls the signal passing through graphene pads into the ground causing attenuation. An independent variation of 5 dB of attenuation is measured along with an independent phase variation of 23 degrees in the frequency range of 4 GHz to 4.5 GHz.

## 1. Introduction

Graphene has been one of the most used innovative materials in recent years [1,2]. Graphene possess exciting electrical properties, apart from its marvelous mechanical, thermal and optical characteristics. Graphene’s high carrier mobility, resulting in high conductance among other semiconductors, makes it suitable for use in a wide variety of electrical applications [2]. One of the most interesting properties of graphene is its variable Fermi energy level with a change in charge density.

This variable Fermi energy level causes a variable conductivity, which is very useful for tunable electrical devices [2]. Conventionally, PIN diodes are used for tunable microwave devices, but they possess a number of drawbacks including larger size and lack of integration. Graphene’s scalable nature, ease of integration, optical transparency, smaller size, higher carrier mobility and higher mechanical strength are some of its distinguishing properties. Additionally, graphene´s variable resistance ensures a very fast switching, unlike MEMS-based switches [3,4,5]. This makes it a better contender for tunable devices. The variation of conductivity with charge density is valid from frequencies ranging from DC up to the millimeter waves, covering the entire microwave frequency range [1].

The synthesis of monolayer graphene requires high technological complexity. Therefore, for the mass scale production of microwave circuitry it is desirable to use multilayered graphene since it exhibits similar tunable conductive behavior at microwave frequencies [2]. It is even more desirable to use commercially available graphene nanoplatelets, since they are very cost-effective and their deployment on circuitry is not technologically demanding.

Several attempts have been made to exploit the variable conductivity for designing tunable microwave components. Attenuators, phase shifters and antennas based on graphene have been proposed in a number of works [6,7,8,9,10,11]. In all of the cases, either the amplitude or the phase was varied, exploiting the tunable conductive behavior of graphene. In the final version of the phase shifter in [11], the number of stubs were increased in order to maximize the phase variation in a graphene-loaded microstrip line. However, it has been noted that the variation of both the amplitude and phase is important to the microwave community for many applications. In quadrature amplitude modulation schemes, the specific variation of the amplitude and phase of the signal is required, in order to complete a set of points on a constellation diagram, each representing a specific amplitude and phase [12,13]. In many beam-forming networks, a specific amplitude and phase is required to excite the individual elements of the network [14,15].

In this paper, for the first time, both the amplitude and phase variations have been introduced on the same transmission line by exploiting graphene’s tunable conductive behavior, unlike only amplitude [7,9,10] or phase [11] variations in previous works. The transmission line is composed of a phase shifter side and an attenuator side, separated from each other by an interdigitated capacitor (IDC). The phase shifter side is composed of a resonating stub and a tapered line connected to grounded vias through graphene pads. The variation of the conductivity of graphene by the application of a voltage bias causes a variation in the phase of the signal on the transmission line. The attenuator side is composed of two pairs of parallel vias, separated from the transmission line by graphene pads. The variation of graphene’s conductivity causes the signal on the transmission line to pass through the graphene into the ground, causing an attenuation. Each side can be independently controlled by a DC bias voltage, resulting in a variation of the amplitude or the phase of the transmitted signal.

## 2. Materials and Methods

### 2.1. Design of the Amplitude Phase Variation Transmission Line

**Figure 1:**
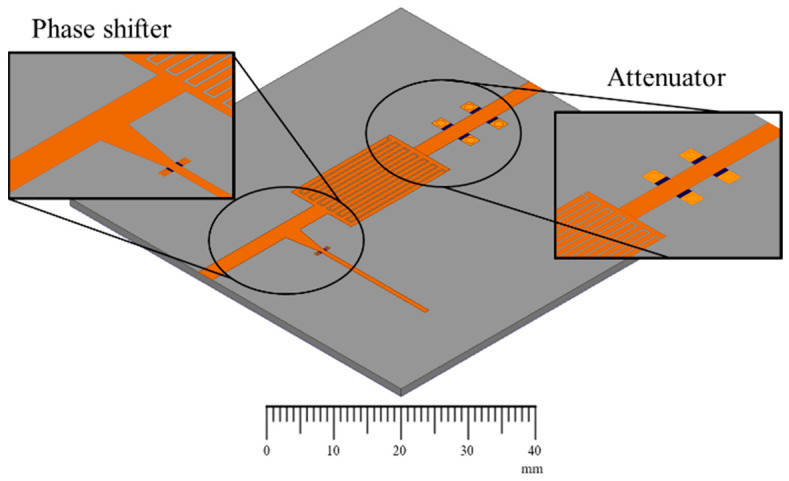
Prototype of the amplitude-phase variation transmission line. In the insets, the black spots are graphene depositions next to grounded vias.

**Figure 2:**
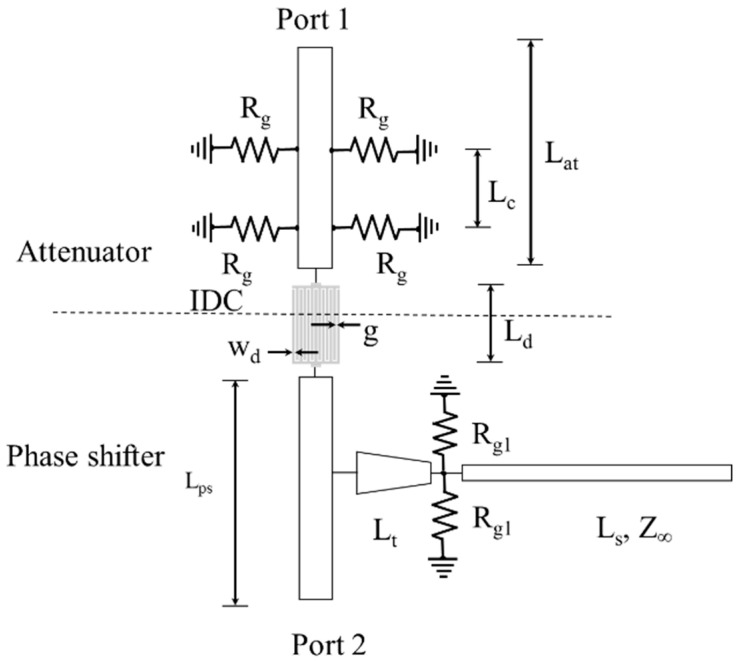
Block diagram of the amplitude-phase variation transmission line with the attenuator coupled to the phase shifter via the IDC. Using different bias voltages, the characteristics of both components are controlled.

The amplitude-phase variation transmission line is composed of a phase shifter, an attenuator and an IDC, as shown in the design of the prototype, shown in Figure 1. A block diagram of the amplitude-phase variation transmission line is shown in Figure 2. A shift in the graphene’s resistance, Rg1, by an applied DC voltage on the phase shifter side increases the reactance introduced in the transmission line. This causes a shift in the phase of the transmitted signal. The lengths of the tapered line and the stub have been optimized for a maximum input reactance variation and a minimum input resistance variation with a change in the graphene’s resistance, Rg1 [10]. On the attenuator side, the transmission line is connected to grounded vias via the graphene pads of resistance Rg. A reduction in the value of Rg caused by the application of a voltage bias causes an increase in the attenuation of the transmitted signal. The two sections of the transmission line are separated by the help of an IDC. This ensures an independent supply of DC voltage to each section.

The amplitude-phase variation transmission line was designed on an FR4 dielectric substrate that has a dielectric constant, Ɛr = 4.38, and a loss tangent, tanδ = 0.02. The thickness of the dielectric substrate is 1.52 mm. The width of the 50 Ω transmission line is 2.9 mm. The IDC is designed to block the DC bias voltage supplied to each section, permitting the RF signal in the frequency band of 3 GHz to 5 GHz.

### 2.2. Finite Element Modelling

The IDC is composed of seven identical fingers on each side. The width of the fingers is wd = 0.75 mm and length is Ld = 19 mm. The distance between the fingers is g = 0.25 mm. On the attenuator side, the length of the transmission line is Lat = 30 mm and the distance between the graphene pads is Lc = 5 mm. On the phase shifter side, the length of the transmission line is Lps = 30 mm and the tapered line is Lt = 6 mm. The length of the stub is Ls = 22.5 mm. The stub is 86 Ω, corresponding to a width of 1 mm. The optimized values of the lengths of the stub and taper can be found in [8], where a detailed analysis for maximizing the reactance variation has been performed. In a similar manner, the lengths of the line sections comprising the attenuator are found in [7].

**Figure 3:**
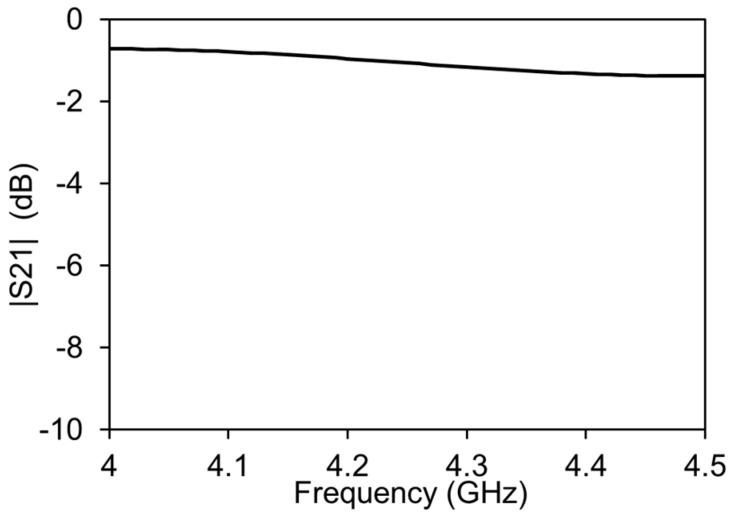
FEM simulations of the IDC without the attenuator and phase shifter.

In order to check the functionality of the IDC and the amplitude-phase variation transmission line, the simulations were performed with a commercial FEM tool Ansys HFSS. The simulation results show that the IDC introduces less than 2 dB of the insertion loss in the system, as shown in Figure 3. This is due to the optimization of the IDC for the least insertion loss in the operating frequency of the amplitude-phase variation transmission line.

For the simulations involving the graphene, the graphene pads were modelled as resistive sheets with assigned resistance values in the range of the estimated resistance of the graphene nanoplatelets. The values of Rg and Rg1 are varied, one at a time, keeping the other at the maximum in order to evaluate the individual impact of the attenuator and phase shifter.

**Figure 4:**
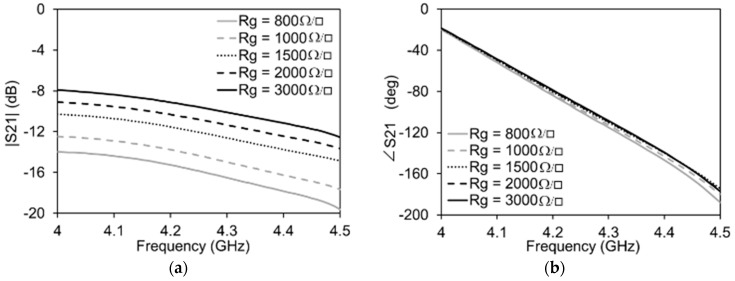
Simulations of the attenuation mode of the transmission line with Rg1 = 3000 Ω/□ for all cases: (**a**) Amplitude variation; (**b**) Phase variation.

**Figure 5:**
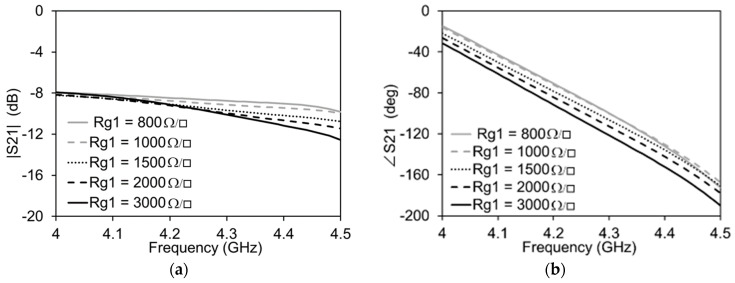
Simulations of the phase shifter mode of the transmission line with Rg = 3000 Ω/□ for all cases: (**a**) Amplitude variation; (**b**) Phase variation.

The maximum and minimum values of the graphene resistance are expected to be 3000 Ω/□ and 800 Ω/□ respectively. It can be clearly seen in Figure 4 that, when the graphene resistance, Rg, pertaining to the attenuator side of the transmission line is varied, the impact on the amplitude is maximum and that on the phase is negligible. The high transmission losses are due to the increased number of vias, resulting in total reduced graphene resistance since all of the graphene pads are in parallel to each other. The contribution of the IDC to the total transmission is less than 1 dB. Similarly, as reported in Figure 5, when the graphene resistance, Rg1, pertaining to the phase shifter side of the transmission line is varied, there is a minimum variation of the amplitude of the signal on the transmission line and a maximum variation of the phase.

### 2.3. Fabrication of the Prototype and Scattering Parameter Measurements

**Figure 6:**
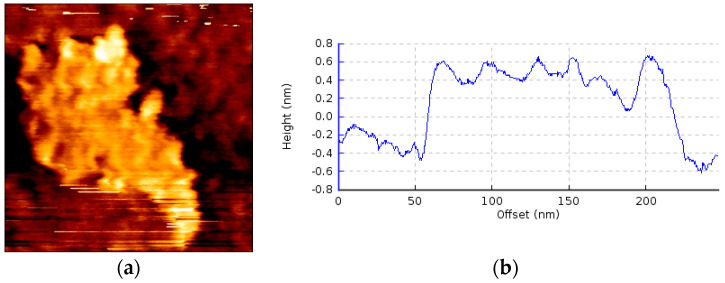
AFM characterization of a graphene flake: (**a**) AFM image with square a size of 250 nm; (**b**) cross-sectional measurement.

**Figure 7:**
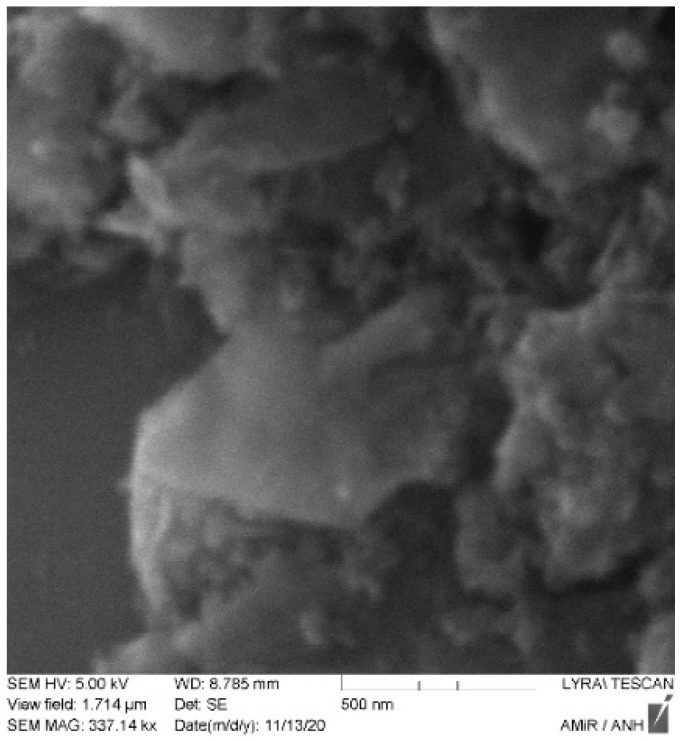
SEM image of the graphene flakes.

The printed circuit board on which the transmission line is present was fabricated with the help of a photolithographic process. The pattern of the transmission line structure is printed with a photoresist on a dual copper-plated dielectric substrate. The unwanted areas lying outside the structure are etched away by ferrous chloride solution. The graphene nanoplatelets used in the transmission line were provided by Alfa Aesar GmbH. The nanoplatelets were characterized by scanning electron microscopy (Tescan Lyra3 FEG) and atomic force microscopy (JPK nanowizard), to investigate their quality and thickness. Figure 6 shows the atomic force microscopy characterization of the multilayered graphene. It can be seen that the flakes in the graphene network are irregularly shaped. In the measurement of the cross-sectional view of the graphene flake (Figure 6b), it can be seen that the thickness of the graphene is equivalent to more than two graphene sheets. An SEM image of the graphene flakes is shown in Figure 7. The measurement was performed with an acceleration voltage of 5 KV. The sharp edges of the graphene flakes are visible in the SEM image. It can be further seen that the graphene flakes form a network in the bunch of the flakes that are to be deposited on the PCB. The graphene nanoplatelets were mixed in isopropyl alcohol and then drop cast in the designated spots on the transmission line. The isopropyl alcohol evaporates, leaving behind the graphene nanoplatelets suspended in the gap, forming a network.

**Figure 8:**
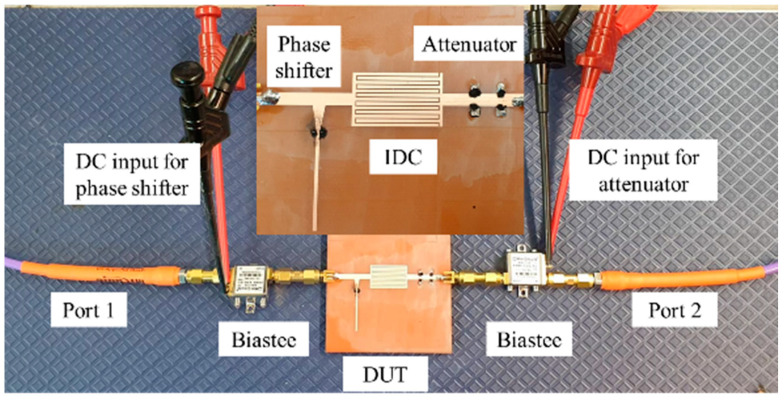
Measurements Setup with device under test in the inset.

The measurement setup of the amplitude-phase variation transmission line is shown in Figure 8. It is composed of a vector network analyzer (Rhode Schwarz ZVA 24) connected to bias tees and the device under test (DUT). The VNA is calibrated at the ends of the bias tees, in order to remove any impact of the bias tees on the measurements.

## 3. Results

**Figure 9:**
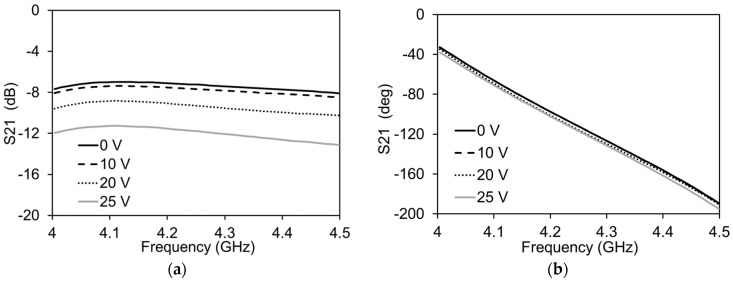
Measurements of the attenuation mode of the transmission line with Vg1 = 0 V for all cases: (**a**) Amplitude variation; (**b**) Phase variation.

The DUT is placed between the two bias tees. The S-parameters are measured for each of the DC bias voltage applied through the bias tees on each side of the transmission line. In this way, the impact of amplitude and phase is individually measured. Table 1 shows the applied DC voltages, currents drawn and the resistances of the graphene pads. It can be seen that in both the attenuator and phase shifter, a reduction in the resistance is caused by an increased supply of a DC voltage. The impact of the variation of the applied DC voltage on the amplitude of S21 is shown in Figure 9. This value of voltage can be related to the respective values of current and resistance shown in Table 1. It can be seen that with an increase in DC voltage, the resistance, Rg, is reduced.

**Figure 10:**
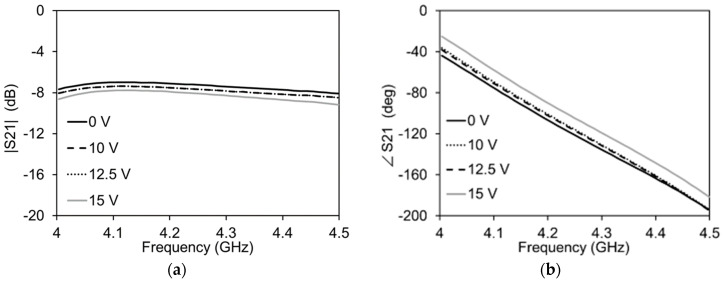
Measurements of the phase shifter mode of the transmission line with Vg = 0 V for all cases: (**a**) Amplitude variation; (**b**) Phase variation.

This causes a large portion of the transmission signal to pass through the graphene into the ground, resulting in attenuation. The maximum variation in |S21| is almost 5 dB, with negligible variation in ∠S21 for the given variation in Rg. Figure 10 shows the variation of the phase and amplitude of S21 when supplying 0 V to the attenuator side (Rg = 3100 Ω). In this case, an increase in the DC voltage reduces Rg1, cutting the stub shorter and reducing the phase of the transmission signal. The total variation in S21 is almost 23 degrees. The variation in the amplitude in this case is 0.42 dB. This results in a figure of merit of 54.76 °/dB, which is comparable to the current state of the art phase shifters reported in Table 2 [16]. The frequency bandwidth of the phase shifter presented here is better than most of the graphene-based phase shifters.

For practical applications regarding the antennas and quadrature amplitude modulation schemes, the demonstrated amplitude and phase variation still need improvement. However, this is a proof of concept, and a first step in acquiring a variable amplitude and phase variation on a single transmission line, exploiting graphene’s tunable resistance. A further increase in the number of stubs can provide a higher phase variation. In a similar manner, an increase in the number of vias on the attenuator side can increase the amplitude variation. The unwanted phase variation (in the attenuator) and amplitude variation (in the phase shifter) can be reduced by optimizing the various lengths and widths of the line sections.

## 4. Discussion

**Figure 11:**
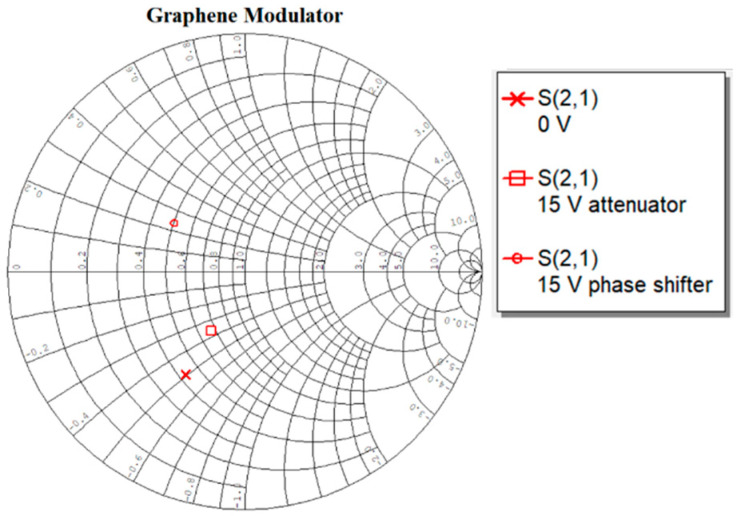
The maximum points of amplitude and phase variation shown on a smith chart.

The benefit of graphene-based amplitude and phase variation is that any number of points can be generated between the extremes by varying the applied DC voltage bias. A small increment is sufficient to vary the graphene resistance, thus varying the amplitude/phase of the signal by a small amount. This could theoretically create infinite number of points on a constellation diagram. In this case of the preliminary modulator, there is no phase shift intended when the attenuation is activated, and no attenuation intended when the phase shifting mode is activated. As shown in Figure 11, moving from the zero position towards the highest phase shift, there is a negligible variation in the amplitude of the signal, whereas in the case of the attenuation, the phase shift is negligible. This shows that the measured data are encouraging but need to be enhanced for increased phase and amplitude variation.

Graphene is a scalable material as it retains the same resistance if the aspect ratio is kept the same with a reduced size. This makes it possible to reduce the power consumption as the variation of amplitude and phase is, in principle tied to the resistance. The maximum power requirement of the phase shifter is almost 255 mW and that of the attenuator is 700 mW. This is on the higher side, but if the device is scaled down, this can be reduced while keeping the aspect ratio and resistance constant. The examples of graphene-based devices on a small scale are reported in [4,5] where the switching speed and scalability is also discussed. A detailed analysis of the heat dissipation resulting from the power consumption will be discussed in a future article.

## 5. Conclusions

A transmission line based on graphene with the capability of amplitude and phase variation is proposed. The transmission line consists of an attenuator side and a phase shifter side, separated through an interdigitated capacitor. This design ensures independent control of the amplitude and phase. The optimized lengths of lines, stubs and vias in the different sections of the line cause attenuation and phase shift by varying graphene’s resistance through an applied DC bias voltage.

## Figures and Tables

**Table 1:** Values of the voltage, current and resistance of the attenuator and phase shifter.

	V_g_ (V)	I_g_ (mA)	R_g_ (Ω)	V_g1_ (V)	I_g1_ (mA)	R_g1_ (Ω)
Attenuator	0	-	3100	0	-	3100
	10	5	2000	0	-	3100
	20	12.5	1600	0	-	3100
	25	28	892	0	-	3100
Phase shifter	0	-	3100	0	-	3100
	0	-	3100	10	5	2000
	0	-	3100	12.5	8	1562
	0	-	3100	15	17	882

**Table 2:** Comparison of the phase shifter performance with state of the art phase shifters.

Technology	Frequency (GHz)	Phase Shift (°)	ΔIL (dB)	FoM (°/dB)	Ref
GFET	3	85	1.3	65.4	[17]
GFET	8	40	-	-	[18]
GFET	30	75	-	-	[18]
GaN HEMT	8–16	22.5	2	11.3	[19]
SiGeHBT	5	170	-	-	[20]
32-nm CMOS	60	175	3.6	48.6	[21]
64-nm CMOS	60	180	3.3	54.54	[22]
130-nm SiGe BiCMOS	60	156	2.2	70.9	[23]
Graphene loaded line	4–4.5	23	0.42	54.7	This work
